# Value of Proximal Femur Geometry in Predicting Occult Hip Fracture

**DOI:** 10.3390/medicina59111987

**Published:** 2023-11-11

**Authors:** Ulf Bökeler, Ulrich Liener, Nils Vogeley, Benjamin Mayer, Cornelia Horsch, Fridolin Tröster, Daphne Eschbach, Steffen Ruchholtz, Tom Knauf

**Affiliations:** 1 Department for Orthopaedics and Trauma Surgery, Marienhospital Stuttgart, Böheimstrasse 37, 70199 Stuttgart, Germany; 2 Department for Epidemiology and Medical Biometry, University of UIm, 89081 Ulm, Germany; benjamin.mayer@uni-ulm.de (B.M.); chorsch@rheinahrcampus.de (C.H.); 3 Department for Diagnostic and Interventional Radiology, Marienhospital Stuttgart, 70199 Stuttgart, Germany; 4 MVZ Hessisch Lichtenau e.v., Kaufungen, 34123 Kassel, Germany; 5 Center for Orthopaedics and Trauma Surgery, University Hospital Giessen and Marburg, 35043 Marburg, Germany; 6 Orthopaedische Klinik Hessisch Lichtenau, 37235 Hessisch Lichtenau, Germany; tknauf89@googlemail.com

**Keywords:** occult proximal femur fracture, fragility fracture, radiographs, cortical thickness, femoral geometry

## Abstract

*Background and Objectives*: Patients with occult hip fractures are a difficult subgroup to treat. MRI is the gold standard for diagnosing occult proximal femur fractures but is costly and may not be readily available in an emergency setting. The purpose of this study was to determine whether changes in the proximal femur geometry can predict the likelihood of an occult hip fracture in patients presenting with hip pain following a ground-level fall. *Material and Methods*: Patients admitted to the hospital with a clinical suspicion of a hip fracture but initial negative radiographs over a seven-year period were included. All patients were additionally investigated with an MRI scan, and retrospectively, six radiologic parameters were obtained on plain radiographs: The cortical thickness index (CTI), the canal to calcar ratio (CCR), the canal flare index (CFI), the morphological cortical index (MCI), the canal bone ratio (CBR) and the canal bone aria ratio (CBAR). Subsequently the result of the plain radiographic indices of the patients with a negative MRI (Group A, no occult fracture) were compared to those with a positive MRI (Group B, occult fracture). *Results*: A total of 78 patients (59 female, 19 male) could be included in the study. The mean age was 82 years. The univariate analyses revealed a poor predictive ability of all radiological parameters with AUC values ranging from 0.515 (CBR) to 0.626 (CTI), whereas a multivariate prognostic model demonstrated improved prognosis (AUC = 0.761) for the CTI (*p* = 0.024), CBAR (*p* = 0.074) and CRR (*p* = 0.081) as the most promising predictive radiological parameters. *Conclusions*: Single radiologic indices obtained from conventional X-rays of the proximal femur have a weak predictive value in detecting occult fractures of the hip and cannot be used as clinical decision-making factors.

## 1. Introduction

The total number of proximal femur fractures is expected to rise significantly in the next decades [1,2]. In the majority of the cases these fractures are easily detected on initial radiographs. The early diagnosis with prompt treatment in conjunction with orthogeriatric management has led to a reduction in mortality and has improved functional results in the past [3]. However, in 2–10% of patients the fracture may not be clearly visible on plain images [4,5]. Patients with these occult fractures are a difficult subgroup to treat. Missing the diagnosis of an occult fracture of the hip femur may lead to secondary displacement and longer hospital stay, whereas immediate diagnosis facilitates prompt operative treatment of the undisplaced fracture, immediate weight bearing and a shorter hospital stay with reduced complications [6].

MRI is the gold standard for diagnosing occult fractures [7,8]. It is thus recommended in an emergency setting whenever it is available [9,10]. However, MRI is costly and may not readily be available in every institution. If available, these patients compete with other emergency patients which are at a higher need for an emergency MRI [11]. Therefore, it would be practical to determine the likelihood of a proximal femur fracture based on changes in the femoral geometry on plain X-rays.

It has been demonstrated that the morphology of the proximal femur is directly related to the likelihood of a proximal femur fracture [12,13] and plays an important role in the evaluation of bone strength [14]. A combination of BMD and radiological measures of upper femur geometry strengthen the informative value of the risk of hip fracture compared to BMD alone [14,15]. Recent studies demonstrate that trabecular bone score (TBS) [16] and hip structural analysis (HSA) examined via dual-energy X-ray absorptiometry (DXA) can predict the risk for proximal femur fractures in postmenopausal women [17]. The assessed morphology on plain radiographs include the thickness of the cortices, the shape and diaphyseal width of the medullary canal and the trabecular pattern of the proximal femur [18,19]. Because radiological indices derived from proximal femur geometry reflect bone quality, these indices can be used to guide the choice for the selection of femoral fixation in total hip arthroplasty [20].

There is a paucity of research on the association of proximal femur geometry with occult proximal femur fractures. Therefore, the aim of this study was to analyze radiologic parameters of the proximal femur taken on plain radiographs to assess whether these parameters can be used as clinical decision-making factors in patients with negative plain radiographs and hip pain following a ground-level fall.

## 2. Materials and Methods

### 2.1. Subjects

After approval by the Institutional Review Board, we conducted an eight-year retrospective search on the hospital information system (Orbis, Dedalus) between 2015 and 2022 which identified all patients which were admitted to the orthopedic trauma department due to immobilizing posttraumatic hip pain. All patients with negative plain X-rays of the pelvis and proximal femur were examined via an MRI of the pelvis and hip to further clarify the cause of the hip pain.

**Figure 1:**
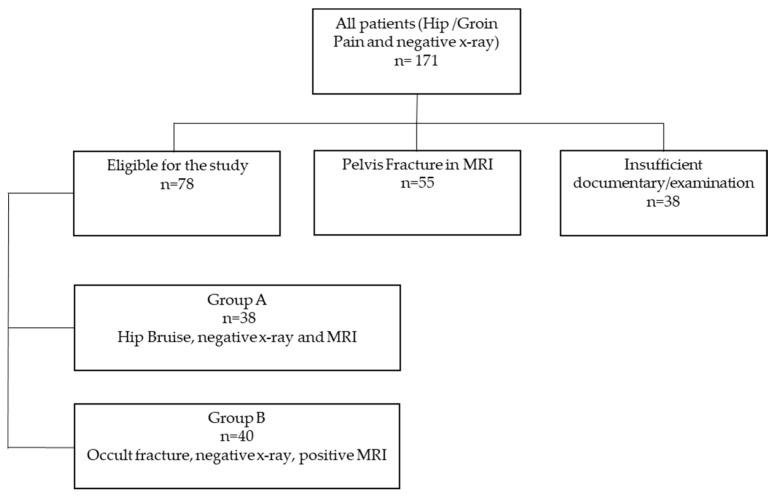
Flowchart.

Of 171 eligible patients, 55 patients had to be excluded because of pelvic fractures, and 38 patients had to be excluded because of insufficient standard X-rays which did not allow the calculation of the radiographic indices. Of the patients that were included in the study, 38 showed no fracture on both X-rays and MRI of the hip (Group A), and in 40 cases, the MRI detected an occult fracture of the proximal femur (Group B) (Figure 1).

From the patient file, the following data were extracted: age, sex and place of residence. Comorbidities and the severity of pre-existing conditions were classified using the Charlson Comorbidity Index (CCI) and the American Society of Anesthesiologists (ASA) risk classification. In addition, vitamin D levels were recorded and categorized.

### 2.2. Radiography and Radiological Indices

Initially, all patients had standard radiographs of the hip (standard ap and axial view) taken. Because of persistent pain, all patients had an additional MRI of the pelvis and hip at a mean of 2.8 days after admission (0–35 days). The a.p. hip radiograph was obtained with the patients in supine position, with the lower extremity rotated 15° internally. The measurements were made on digital radiographs using the IMPAX system (Agfa, Germany).

**Figure 2:**
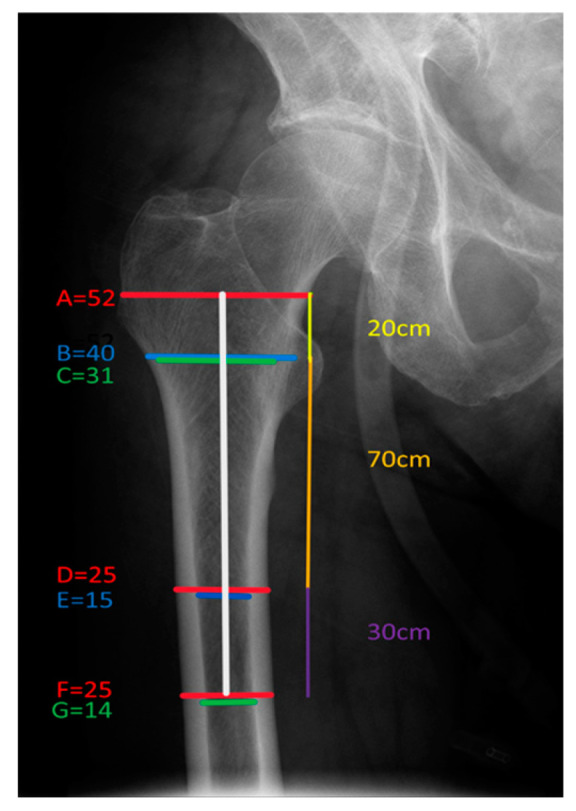
Illustration of measurement methods of six indices on the anteroposterior (AP) hip radiograph. CTI = (F − G)/F; CCR = G/C; CFI = A/G; MCI = B/E; CBR = G/F; CBAR = (E + G)/(D + F).

Proximal femur morphology was assessed with 6 different radiological indices (Figure 2): the cortical thickness index (CTI) [20], the canal to calcar ratio (CCR) [20,21], the canal flare index (CFI) [22], the morphological cortical index (MCI) [23], the canal bone ratio (CBR) [24] and the canal bone aria ratio (CBAR) [25]. Measurements were taken independently by senior orthopedic surgeon and a 4th-year orthopedic resident.

### 2.3. MRI

**Figure 3:**
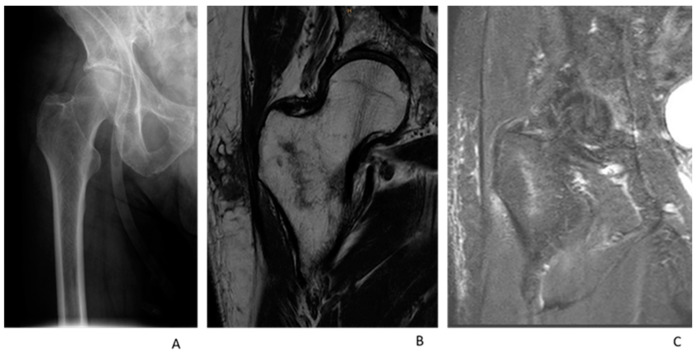
An 85-year-old man with severe hip pain after a ground-level fall. (**A**): AP Radiograph showing no fracture; (**B**): T1 Coronar Sequence MRI showing intertrochanteric fracture line; (**C**): STIR Coronar Sequence showing intertrochanteric oedema.

**Figure 4:**
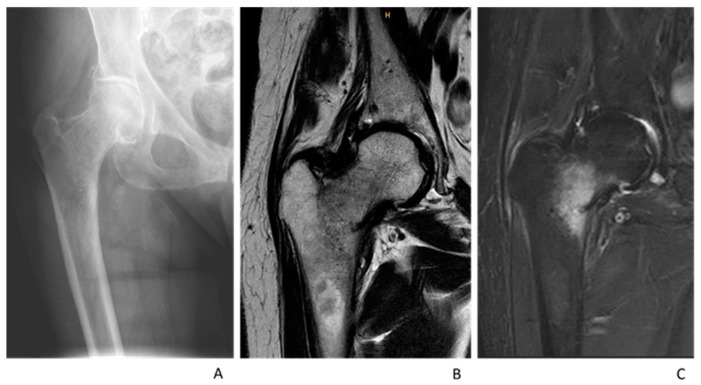
An 87-year-old woman with immobilizing hip pain after low-energy fall. (**A**): AP Radiograph showing no fracture; (**B**): T1 Coronar Sequence MRI lateral femoral neck fracture line; (**C**): STIR Coronar Sequence showing lateral femoral neck oedema.

For the MRI, a standardized institutional trauma pelvis/hip protocol using a T1-weighted turbo-spin echo axial and coronal + STIR (short-tau inversion recovery) coronal + T2 mDIXON (modified Dixon) axial scan using a Philips 3T or a Philips 1.5T. All MRIs were reviewed by a senior radiologist. A hip fracture was diagnosed when there was evidence of trabecular oedema of intermediate signal and a low intensity signal traversing the bone on the T1 sequence with a corresponding high signal traversing the bone on the STIR sequence (Figure 3 and Figure 4). When in doubt, the T2 mDIXON sequence was used for further clarification and the detection of fracture lines.

### 2.4. Statistical Analysis

All analyses were performed using the R software for statistical computing (version 4.2.2). Demographic patient data were descriptively analyzed using frequencies in case of categorical data. Continuous data were presented using arithmetic mean, standard deviation (SD), median and range. Reliability of radiological parameters assessed by two independent raters was checked by means of intra-class correlation coefficient (ICC) as well as Bland–Altman analysis (psych and blandr packages in R). Receiver operating characteristic (ROC) analysis was performed in order to check the prognostic value of the radiological parameters in light of their ability to predict the presence of occult hip fracture. Since reliability analysis revealed acceptable agreement between both raters for all investigated parameters with no presence of bias in Bland–Altman analysis, the arithmetic mean of both independent ratings for each patient was used for the ROC analyses. Sensitivity, specificity, area under the curve (AUC), all ranging from 0 to 1 (the higher the better), and optimal cut-point using Youden’s index was calculated using the *Optimal Cutpoints* package in R. A multivariable logistic regression model was finally used to build a multivariable prognostic model including a dichotomous version of all radiological parameters checked in ROC analyses. A *p*-value < 0.05 was considered statistically significant. 

## 3. Results

Of the 40 patients with a proximal femur fracture, 28 patients had an intertrochanteric fracture and 12 a cervical neck fracture; 22 (78%) of the intertrochanteric fractures were treated surgically with an intermedullary nail (Gamma3 Locking Nail, Stryker^®^); in 3 (11%) cases, the fracture was stabilized by a Dynamic Hip Screw (DMS Martin^®^); and 3 (11%) patients were treated conservatively.

The occult cervical neck fractures were stabilized in 7 (59%) cases using a Dynamic Hip Screw (DMS Martin^®^), 3 (25%) fractures were treated operatively with a cemented bipolar hip prothesis (Avenir, Zimmer^®^), 1 (8%) with a total hip replacement (Avenir, Zimmer^®^) and 1 patient (8%) was treated conservatively. All patients which were treated conservatively refused an operation and were treated with restricted weight bearing for 6 weeks. All patients were referred to our fracture liaison service for osteoporosis evaluation and treatment.

Overall, 59 female (76%) and 19 male (24%) patients were included in the study. The mean age of the MRI fracture negative patients was 84.3 years (SD 6.0 years.) and 79.2 years (SD 11.6 years) in occult hip fracture patients, respectively. The demographic and medical related data are shown in Table 1. Only gender distribution demonstrated a significant difference between both groups (*p* = 0.001).

### 3.1. Reliability of Measurements

Bland–Altman analysis revealed no relevant bias associated with the two ratings of both independent physicians for all six radiological variables, i.e., all 95% confidence intervals for the mean difference in both ratings included 0. Accordingly, the ICC values for all six radiological parameters tested were larger than 0.68, which could be referred to acceptable reliability, except for CBAR (ICC = 0.48) and CRR (ICC = 0.43), where ICC-related reliability was poor. The mean values of the assessed radiological parameters are shown in Table 2.

### 3.2. Results of ROC Curve Analysis

Overall, the univariate analyses revealed a poor predictive ability of all radiological parameters with AUC values ranging from 0.515 (CBR) to 0.626 (CTI). The latter showed a sensitivity of 0.854 and a specificity of 0.417. There was in general a tendency of insufficient specificity, which is likely to cause the poor AUC values.

A multivariable logistic regression model enabling a ROC analysis based on multiple predictor variables was implemented as follows. First, univariate ROC analysis was conducted based on each of the geometric variables (CTI, CFI, MCI, CBR, CBAR, CRR) using the *cutpointr* package in R (version 4.2.2). These analyses revealed univariate results on area under the curve (AUC) and optimal cut-off values, which could be used to dichotomize patients in rather occult hip fracture or hip bruise patients (Table 3).

**Figure 5:**
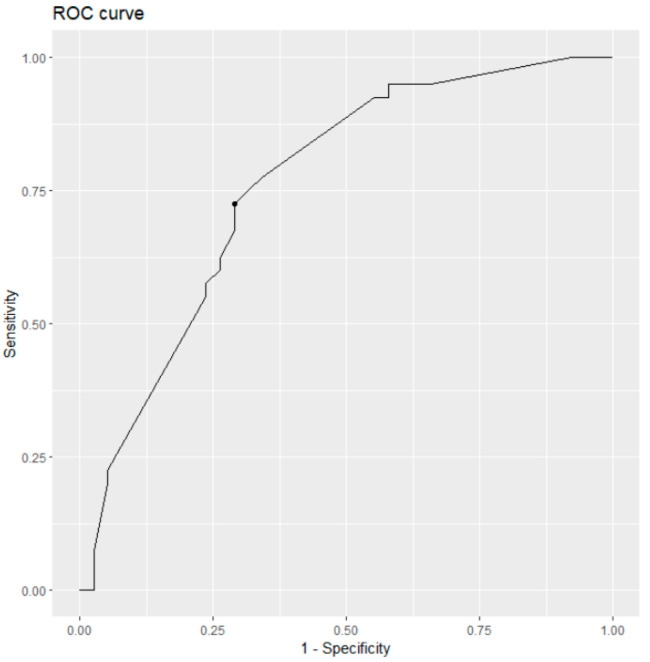
Multivariable prognosis of occult hip fracture events.

The calculated optimal cut-points from univariate analyses for each of the six radiological parameters (CTI: 35.39, CFI: 3.96, MCI: 2.59, CBR: 0.49, CBAR: 0.47, CRR: 0.55) were used to dichotomize the original measurements, and a multivariate prognostic model was set up to provide an estimation model for a patient’s individual likelihood to have an occult hip fracture (Figure 5). This model demonstrated improved prognosis (AUC = 0.761) with CTI (*p* = 0.024), CBAR (*p* = 0.074) and CRR (*p* = 0.081) as the most promising predictive radiological parameters.

## 4. Discussion

Hip pain is a common complaint following low-velocity or ground-level falls in geriatric patients. The pain can either be caused by a soft tissue trauma, pelvic or hip fracture. Soft tissue trauma and pelvic fractures are usually treated conservatively, whereas early diagnosis and consecutive treatment in hip fractures is important because the mortality and complication rate increase if operative treatment is delayed greater than 24 h [6,26]. Given the high morbidity and mortality associated with hip fractures, it is important that these patients are identified.

The prevalence of occult hip and pelvic fractures has been estimated to be approximately 10% in patients requiring a radiographic examination after a fall [4,27]. The prevalence of hip fracturs in the cohort of occult fractures does vary. In larger studies, it has been reported to be up to 92% [4]. Most of the hip fracture patients fall into the typical age group aged over 70 year; nevertheless, there are reports of younger patients as well [27]. It is therefore important to be suspicious if a patient presents with negative conventional X-rays and persistent pain and an inability to weight bear regardless of age.

MRI has been shown to have a higher sensitivity and specificity in diagnosing intertrochanteric fractures compared to plain radiographs and CT scanning and is therefore regarded as the gold standard [28]. MRI is costly, requires experience and may not be readily available in every institution at any given time.

CT scans are readily available, but especially for occult intertrochanteric fractures, they are inferior in detecting the fracture [28,29]. Dual-energy CT is a promising technique, but its diagnostic accuracy does not equal the accuracy of MRI [30]. A study of Reddy et al. demonstrated a sensitivity of 90% and specificity of 40% for nondisplaced femoral neck fractures in dual-energy CT [31].

Dual-energy X-ray absorptiometry (DXA) is considered to be the gold standard for measuring bone mineral density (BMD) over the proximal femur. However, because DEXA may not be readily available in every institution, several authors have proposed that changes in the femoral geometry on plain radiographs could be used to assess the bone quality of the proximal femur. The assessed morphology includes the thickness of the cortices, the shape and diaphyseal width of the medullary canal, and the trabecular pattern of the proximal femur [18,19]. Based on these measurements, indices can be calculated to reflect the bone quality of the proximal femur. Initially these indices were proposed to guide the choice for the selection of femoral fixation in total hip arthroplasty [20].

The aim of this study was to assess whether the proximal femur morphology on plain radiographs of patients with immobilized hip pain after a low-energy fall can be used as a clinical decision-making factor.

In our study, we used indices that measure cortical thickness (CBR, CTI), the morphology, e.g., champagne flute (CCR, CFI), and indices that combine both morphology and cortical thickness of the proximal femur (MCI, CBAR).

Yeung et al. were the first to demonstrate a strong correlation of the CBR and the MCI with the T score measured in DEXA scans [24]. In addition, CBR and MCI showed the best overall performance in ROC curves for diagnosing osteoporosis when compared to CCR and CFI [24]. They defined a CBR ratio of 0.49 or higher as a cut off for osteoporosis. Using the cut off Yeung et al. [24] introduced, in our patient population, 62.5% of patients in the occult fractures would be classified as osteoporotic and 47.4% of the patients in the MRI fracture negative group.

The findings that proximal femur morphology is strongly associated with bone mineral density has been confirmed by other studies [32]. Sah et al. demonstrated that isolated cortical morphology measured as cortical thickness index (CTI) showed a correlation with T scores, whereas the morphology of the proximal femur (CCR) did not. Given these results, the authors concluded that the femoral diaphysis may best reflect overall bone integrity based on both geometry and structure.

Several studies assessed risk factors for proximal femur fractures. Hans et al., with data derived from DXA images, showed that trabecular bone score (TBS) improves fracture risk prediction and can be incorporated to the Fracture Risk Assessment tool (FRAX^®^) to enhance fracture prediction [16]. Hip structural analysis (HAS), also performed via DXA, analyzes variables related to proximal hip geometry such as the cross-sectional area (CSA), cross-sectional moment of inertia (CSMI), section modulus (Z) and buckling ratio (BR) [33,34]. LaCroix et al. demonstrated that a high BR and low values of CSA, CSMI and Z are associated with poor hip strength and a higher tendency to fracture [35]. A latest study by Johnsen et al. pointed out that impaired parameters of proximal hip geometry and a low trabecular bone score are potential risk factors for osteoporotic fractures of the femoral neck in postmenopausal women [17].

Because of the proven relationship between femur morphology and BMD, we sought to analyze if these changes could be used to determine the likelihood of fracture. Since the relationship between proximal femur geometry and occult fractures has not been investigated so far, we measured and calculated the ratios irrespective of their proven correlation with BMD.

Taken individually, each of the six parameters showed a weak predictive ability to detect occult fractures of the hip. The predictive accuracy was significantly increased when all the six parameters were included simultaneously in a multivariable model. This model enables researchers to calculate a patient’s propensity score P(E = 1|X = x) = [exp(β_0_ + β_1_X_1_ + … + β_p_X_p_)/1 + exp(β_0_ + β_1_X_1_ + … + β_p_X_p_)] to have an occult hip fracture (E = 1) based on given covariates (X = x). In particular, based on the six dichotomized radiological parameters (using the above-mentioned cut-off values for each), the final equation for calculating the probability of an occult hip fracture event was P(E = 1|X = x) = −0.14 + 1.88∙CTI_cut + 0.12∙CFI_cut − 1.26∙MCI_cut − 0.97∙CBR_cut + 1.75∙CBAR_cut − 1.05∙CRR_cut. This formula might be an additional tool to predict the risk of a occult fractures. The reason why each single parameter does not predict patients at risk of an occult fracture could be explained by the low BMD of our patient population, with most of the patients belonging to a high-risk group with low BMD.

### Limitations

This is a retrospective study and there is always the risk of selection bias by not identifying patients in our institutions electronic records or the radiological database. The interobserver reliability was satisfactory and showed more beneficial results compared to the study by Yeung et al. [24] but we could not reach out to the excellent results presented by Nguyen et al. [36]. As with other studies on the same topic, we are unable to account for the possibility that patients were improperly classified as positive or negative for hip fractures in the initial radiographs [7].

## 5. Conclusions

In summary, there is abundant evidence in the literature that proximal femur geometry is correlated to bone mineral density. Bone mineral density is recognized as a key determinate of fracture risk. Microarchitectural alterations and geometric changes are also proven to contribute significantly to fracture risk in fragility fractures of the hip.

To our knowledge, this is the first study which analyzes proximal femur geometry in patients with occult proximal femur fractures. We could demonstrate that single parameters of proximal femur morphology cannot be used as a clinical decision-making factor when deciding whether additional diagnostics are indicated.

## Figures and Tables

**Table 1:** Patient characteristics.

	Group A (n = 38)	Group B (n = 40)
Age (Mean (SD))	84.3 (6.0)	79.2 (11.6)
Gender n (%)		
- Female	35 (92%)	24 (60%)
- Male	3 (8%)	16 (40%)
Residence n (%)		
- Independent	23 (61%)	12 (30%)
- With help at home	6 (16%)	20 (50%)
- Nursing home	7 (18%)	6 (15%)
- Hospital or rehab clinic	2 (5%)	2 (5%)
ASA Grade n (%)		
- ASA 1	-	-
- ASA 2	2 (5%)	1 (2%)
- ASA 3	36 (95%)	35 (88%)
- ASA 4	-	4 (10%)
CCI (median (range))	5 (3–9)	6 (2–11)
MRI days after admission (median (range))	2 (0–14)	1.5 (0–35)
Vitamin D (mg/mL) n		
- <12	14	14
- 13–20	5	9
- 21–30	6	6
- >30	13	9
- No data	5	2

**Table 2:** Description of geometric variables.

Variable	Hip Bruise (n = 38)		Occult Proximal Femur Fracture (n = 40)	
[mean (SD)]	Rater 1	Rater 2	Rater 1	Rater 2
CTI	31.1 (3.68)	31.3 (3.13)	32.6 (3.63)	32.3 (4.33)
CFI	3.7 (0.74)	3.6 (0.75)	3.5 (0.65)	3.4 (0.73)
MCI	2.6 (0.57)	2.5 (0.51)	2.4 (0.37)	2.4 (0.39)
CBR	0.5 (0.07)	0.5 (0.09)	0.5 (0.08)	0.5 (0.07)
CBAR	0.5 (0.08)	0.5 (0.09)	0.5 (0.07)	0.6 (0.15)
CRR	0.5 (0.12)	0.5 (0.14)	0.5 (0.11)	0.5 (0.10)

**Table 3:** Analyses of univariate analysis.

Variable	Univariate Analysis	
	AUC	Optimal Cut-Off
CTI	0.580	35.39
CFI	0.565	3.96
MCI	0.607	2.60
CBR	0.568	0.49
CBAR	0.630	0.47
CRR	0.563	0.55

## Data Availability

The datasets used and/or analyzed during the present study are available from the corresponding author on reasonable request.
